# Supplementary material for: Rejuvenating conventional dendritic cells and T follicular helper cell formation after vaccination
Source: eLife. 2020 Mar 24;9:e52473. doi: 10.7554/eLife.52473 (PMC7093110; doi:10.7554/eLife.52473)
Supplement: Supplementary file 1. [file elife-52473-supp1.docx]

**Supplementary Table S1: Key Resource Table.**

| **Key Resources Table** | | | | |
| --- | --- | --- | --- | --- |
| **Reagent type (species) or resource** | **Designation** | **Source or reference** | **Identifiers** | **Additional information** |
| strain, strain background (*Mus musculus*) | C57BL/6 | JAX/Babraham Breeding Unit | C57BL/6Babr |  |
| strain, strain background (*Mus musculus,*C57BL/6) | *Ifnar1^-/-^* | U. Kalinke  doi:10.1038/nature10163 | *Ifnar1^tm2a(EUCOMM)Wtsi^*  RRID:MGI:5527593 |  |
| strain, strain background (*Mus musculus,*C57BL/6) | *Ifnar1^fl/fl^* | U. Kalinke  doi:10.4049/jimmunol.176.4.2074 | *Ifnar1^tm1Uka^*  RRID:MGI:2655303 |  |
| strain, strain background (*Mus musculus,*C57BL/6) | *Cd11c^cre^* | R.Roychoudhuri  doi:10.1084/jem.20062648 | *B6.Cg-Tg(Itgax-cre)1-1Reiz/J*  RRID: MGI:3763375 |  |
| strain, strain background (*Mus musculus,* C57BL/6) | OTII TCR | K. Okkenhaug  doi:10.1046/j.1440-1711.1998.00709.x | *(TCR)OT2*  RRID:MGI:3046083 |  |
| strain, strain background (*Mus musculus,*C57BL/6) | TCR7 TCR | A. O’Garra  doi:10.1002/eji.200636432 | *Tg(TcraBO4H9.1)7Aog*  RRID:MGI:5603581 |  |
| strain, strain background (*Mus musculus,*C57BL/6) | *H2^+/-^* | A. Liston  doi:10.1073/pnas.96.18.10338 | B6.129S2-*H2^dlAb1-Ea^*/J  RRID: MGI:2174354 |  |
| Dye | eFluor780 Viability dye | eBioscience | 65-0865-14 | (1:5000) |
| antibody | APC-eFluor780-coupled anti-human CD14  Mouse monoclonal | eBioscience | Clone:61D3  RRID:AB_1272044 | (1:50) |
| antibody | APC-eFluor780-coupled anti-human CD16  Mouse monoclonal | eBioscience | Clone:eBioCB16  RRID:AB_11220086 | (1:50) |
| antibody | APC-eFluor780-coupled anti-human CD19  Mouse monoclonal | eBioscience | Clone:HIB19  RRID:AB_1582231 | (1:50) |
| antibody | BUV395-coupled anti-human CD3  Mouse monoclonal | BD Biosciences | Clone:UCHT1  RRID:AB_2744387 | (1:100) |
| antibody | PerCp-Cy5.5-coupled anti-human CD4  Mouse monoclonal | BD Biosciences | Clone:RPA-T4  RRID:AB_1727476 | (1:50) |
| antibody | BUV737-coupled anti-human CD45RA  Mouse monoclonal | BD Biosciences | Clone:HI100  RRID:AB_2819204 | (1:25) |
| antibody | PE-Cy7-coupled anti-human PD1  Mouse monoclonal | eBioscience | Clone:eBioJ105  RRID:AB_10853032 | (1:25) |
| antibody | BB515-coupled anti-human CXCR5  Rat monoclonal | BD | Clone:RF8B2  RRID:AB_2738871 | (1:25) |
| antibody | BV421-coupled anti-mouse Bcl6  Mouse monoclonal | BD Biosciences | Clone:K112-91  RRID:AB_2738159 | (1:100) |
| antibody | PE-Cy7-coupled anti-mouse Bcl6  Mouse monoclonal | BD Biosciences | Clone:K112-91  RRID:AB_2738292 | (1:100) |
| antibody | PE-Cy7-coupled anti-mouse CD95  Armenian hamster monoclonal | BD Biosciences | Clone:Jo2  RRID:AB_396768 | (1:200) |
| antibody | BV605-coupled anti-mouse IgG1  Rat | BD Biosciences | Clone:A85-1  RRID:AB_2738116 | (1:100) |
| antibody | BUV395-coupled anti-mouse CD3  Armenian hamster monoclonal | BD Biosciences | Clone:145-2C11  RRID:AB_2738278 | (1:300) |
| antibody | PE-Cy7-coupled anti-mouse CD3  Armenian hamster monoclonal | BD Biosciences | Clone:145-2C11  RRID:AB_394460 | (1:300) |
| antibody | BUV395-coupled anti-mouse CD19 Rat monoclonal | BD Biosciences | Clone:1D3  RRID:AB_2722495 | (1:200-1:300) |
| antibody | PE-Cy7-coupled anti-mouse CD19 Rat monoclonal | BD Biosciences | Clone:1D3  RRID:AB_394495 | (1:200-1:300) |
| antibody | PE-Cy7-coupled anti-mouse B220  Rat monoclonal | BD Biosciences | Clone:RA3-62B  RRID:AB_394458 | (1:200-1:400) |
| antibody | BUV395-coupled anti-mouse B220  Rat monoclonal | BD Biosciences | Clone:RA3-62B  RRID:AB_2738427 | (1:200-1:400) |
| antibody | BV786-coupled anti-mouse B220  Rat monoclonal | BD Biosciences | Clone:RA3-62B  RRID:AB_2738472 | (1:200-1:400) |
| antibody | BUV395-coupled anti-mouse CD8a  Rat monoclonal | BD Biosciences | Clone:53-6.7  RRID: AB_2732919 | (1:200) |
| antibody | AF647-coupled anti-mouse CD64  Mouse monoclonal | BD Biosciences | Clone:X54-5/71  RRID:AB_647120 | (1:200) |
| antibody | PE-Cf594-coupled anti-mouse CD11b  Rat monoclonal | BD Biosciences | Clone:M1/70  RRID:AB_11154422 | (1:200) |
| antibody | BV786-coupled anti-mouse CD103  Armenian hamster monoclonal | BD Biosciences | Clone:M290  RRID:AB_2738744 | (1:200) |
| antibody | PE-coupled anti-mouse CD86  Rat monoclonal | BD Biosciences | Clone:GL1  RRID:AB_394994 | (1:300) |
| antibody | BV510-coupled anti-mouse CD86  Rat monoclonal | BD Biosciences | Clone:GL1  RRID:AB_2737991 | (1:300) |
| antibody | APC-AF780-coupled anti-mouse PD1  Armenian Hamster monoclonal | eBioscience | Clone:J43  RRID:AB_2574001 | (1:200) |
| antibody | APC-coupled anti-mouse Foxp3  Rat monoclonal | eBioscience | Clone:FJK-16S  RRID:AB_469457 | (1:100-1:200) |
| antibody | AF488 coupled anti-mouse Ki67  Rat monoclonal | eBioscience | Clone:SolA15  RRID:AB_2802330 | (1:100) |
| antibody | AF700-coupled anti-mouse Ki67  Rat monoclonal | eBioscience | Clone:SolA15  RRID:AB_2637480 | (1:100) |
| antibody | Biotin-coupled anti-mouse Gr1  Rat monoclonal | eBioscience | Clone:RB6-8C5  RRID:AB_466800 | (1:200) |
| antibody | eF450-coupled anti-mouse CD38  Rat monoclonal | eBioscience | Clone:90  RRID:AB_11218302 | (1:400) |
| antibody | PerCp-Cy5.5-coupled anti-mouse CD172a  Rat monoclonal | Biolegend | Clone:P84  RRID:AB_2563547 | (1:200) |
| antibody | eF450-coupled anti-mouse CD24  Rat monoclonal | eBioscience | Clone:M1/69  RRID:AB_1311169 | (1:500) |
| antibody | APC-AF780-coupled anti-mouse CD11c  Armenian hamster monoclonal | eBioscience | Clone:N418  RRID:AB_1548652 | (1:200) |
| antibody | APC-coupled anti-mouse CD11c  Armenian hamster monoclonal | eBioscience | Clone:N418  RRID:AB_469346 | (1:200) |
| antibody | APC-coupled anti-mouse CD80  Armenian hamster monoclonal | eBioscience | Clone:16-10A1  RRID:AB_469417 | (1:300) |
| antibody | PE-Cy5-coupled anti-mouse CD80  Armenian hamster monoclonal | eBioscience | Clone:16-10A1  RRID:AB_468774 | (1:300) |
| antibody | PerCp-Cy5.5-coupled anti-mouse CD45.2  Mouse monoclonal | eBioscience | Clone:104  RRID:AB_953590 | (1:200) |
| antibody | AF700-coupled anti-mouse MHC-II  Rat monoclonal | eBioscience | Clone:M5/114.12.2  RRID:AB_494009 | (1:400) |
| antibody | BV421-coupled anti-mouse CXCR5  Rat monoclonal | Biolegend | Clone:L138D7  RRID:AB_2562127 | (1:100) |
| antibody | V500 -coupled anti-mouse CD4  Rat monoclonal | Biolegend | Clone:RM4-5  RRID:AB_1937315 | (1:400-1:800) |
| antibody | PE-coupled anti-mouse CD4  Rat monoclonal | Biolegend | Clone:RM4-5  RRID:AB_313690 | (1:400-1:800) |
| antibody | BV605-coupled anti-mouse CD4  Rat monoclonal | Biolegend | Clone:RM4-5  RRID:AB_2800581 | (1:400-1:800) |
| antibody | PerCp-Cy5.5-coupled anti-mouse CD44  Rat monoclonal | Biolegend | Clone:IM7  RRID:AB_2076206 | (1:200) |
| antibody | AF488-coupled anti-mouse GL7  Rat monoclonal | Biolegend | Clone:GL7  RRID:AB_2563284 | (1:100) |
| antibody | PE-coupled anti-mouse F4/80  Rat monoclonal | Biolegend | Clone:BM8  RRID:AB_893498 | (1:200) |
| antibody | BV605-coupled anti-mouse F4/80  Rat monoclonal | Biolegend | Clone:BM8  RRID:AB_2562305 | (1:200) |
| antibody | BV650-coupled anti-mouse XCR1  Mouse monoclonal | Biolegend | Clone:ZET  RRID:AB_2566410 | (1:200) |
| antibody | PE-Cy7-coupled anti-mouse CD40  Rat monoclonal | Biolegend | Clone:3/23  RRID:AB_10933422 | (1:300) |
| antibody | AF700-coupled anti-mouse CD45.1  Mouse monoclonal | Biolegend | Clone:A20  RRID:AB_493732 | (1:200) |
| antibody | PerCp-Cy5.5-coupled anti-mouse PDCA-1  Rat monoclonal | Biolegend | Clone:927  RRID:AB_2566646 | (1:100) |
| antibody | PE-coupled anti-mouse TCR Vβ3  Armenian hamster monoclonal | BD Biosciences | Clone:KJ25  RRID:AB_394709 | (1:100) |
| antibody | APC-coupled anti-mouse TCR Vα2  Rat monoclonal | eBioscience | Clone:B20.1  RRID:AB_1659733 | (1:100) |
| Labelling agent | APC/PE-Cy7/BV510-coupled Streptavidin | Biolegend | #Cat 405207  #Cat 405206  #Cat 405233 | (1:600) |
| antibody | Biotin-coupled anti-mouse Eα 52-68 peptide  Mouse monoclonal | eBioscience | Clone:eBioY-Ae  RRID:AB_657822 | (1:200) |
| antibody | PE-coupled 1W1K-IAb tetramer | NIH Tetramer core facility | PE-coupled “I-A(b) EAWGALANKAVDKA” | (1:100) |
| antibody | PE-coupled anti-mouse pSTAT1  Mouse monoclonal | BD Biosciences | Clone:pY701  RRID:AB_399855 | (1:10) |
| antibody | eF450-conjugated anti-mouse Foxp3 Rat monoclonal | Thermo Fisher Scientific | Clone: FJK16S  RRID:AB_1518812 | IF: (1:50) |
| antibody | A647 anti-mouse CD3ε Syrian hamster monoclonal | Thermo Fisher Scientific | Clone:500A2  RRID:AB_2536582 | IF: (1:200) |
| antibody | anti-mouse Ki67 Rabbit polyclonal | Abcam | #Cat:15580  RRID:AB_443209 | IF: (1:100) |
| antibody | AF647-conjugated anti-mouse IgD  Rat monoclonal | Biolegend | Clone:11-26c.2a  RRID:AB_893529 | IF: (1:100) |
| antibody | AF568-conjugated anti-hamster IgG Goat polyclonal | Life Technologies | #Cat:A-21112  RRID:AB_2535761 | IF: (1:500) |
| antibody | AF488-conjugated anti-rabbit IgG  Goat polyclonal | Abcam | #Cat:150077  RRID:AB_2630356 | IF: (1:400) |
| sequence-based reagent | *Ifnb1* primers | Thermo Fisher Scientific | #Cat:4331182, Mm00439552_s1 | RT-qPCR TaqMan probe/primer set |
| sequence-based reagent | *Mx1* primers | Thermo Fisher Scientific | #Cat:4331182, Mm00487796_m1 | RT-qPCR TaqMan probe/primer set |
| sequence-based reagent | *Ifit1* primers | Thermo Fisher Scientific | #Cat:4331182, Mm00515153_m1 | RT-qPCR TaqMan probe/primer set |
| sequence-based reagent | *Hprt* primers | Thermo Fisher Scientific | #Cat:4331182, Mm03024075_m1 | RT-qPCR TaqMan probe/primer set |
| peptide, recombinant protein | recombinant murine IFNα | PBL assay science | #Cat:12105-1 |  |
| peptide, recombinant protein | HEL | Sigma | #Cat:62970 | Lysozyme from chicken egg white |
| peptide, recombinant protein | OVA | Sigma | #Cat:A5503 | Albumin from chicken egg white |
| peptide, recombinant protein | 1W1K-biotin | Cambridge Research Biochemicals | Peptide sequence:”biotin-GSGEA-W-GALANKA-V-DKA-acid” | custom-made |
| commercial assay or kit | SMART-Seq v4 Ultra Low Input RNA Kit for Sequencing | Clontech | #Cat:634890 |  |
| commercial assay or kit | Nextera XT kit | Illumina | #Cat:FC-131-1096 |  |
| commercial assay or kit | pan dendritic cell isolation kit | Miltenyi | #Cat:130-100-875 |  |
| commercial assay or kit | RNeasy Micro kit | Qiagen | #Cat:74004 |  |
| commercial assay or kit | RNeasy Mini kit | Qiagen | #Cat:74104 |  |
| chemical compound, drug | IFNAR1-blocking antibody  Mouse monoclonal | BioXCell | #Cat:BE0241  RRID:AB_2687723 |  |
| chemical compound, drug | Pertussis toxin | Sigma | #Cat:P7208 |  |
| chemical compound, drug | NP-KLH | Biosearch Technologies | #Cat:N-5060-25 |  |
| chemical compound, drug | NP-OVA | Biosearch Technologies | #Cat:N-5051-100 |  |
| chemical compound, drug | NP-e-Aminocaproyl-OSu | Biosearch Technologies | #Cat:N-1021-100 |  |
| chemical compound, drug | Streptavidin | Sigma | #Cat:S4762-10MG |  |
| software, algorithm | Prism | GraphPad | v6/v7  RRID:SCR_002798 |  |
| software, algorithm | FlowJo | Tree Star | v10  RRID:SCR_008520 |  |
| software, algorithm | ImageJ | NIH  doi:10.1038/nmeth.2089 | RRID:SCR_003070 |  |
| software, algorithm | SeqMonk software package | Babraham Institute  Source:https://www.bioinformatics.babraham.ac.uk/projects/seqmonk/ | RRID:SCR_001913 |  |
| software, algorithm | Trim Galore v0.4.2 | Babraham Institute  Source:https://www.bioinformatics.babraham.ac.uk/projects/trim_galore/ | RRID:SCR_011847 |  |
| software, algorithm | HISAT2 | John Hopkins University CCB  doi:10.1038/nmeth.3317 | RRID:SCR_015530 |  |
| software, algorithm | Rsubread | Bioconductor  doi:10.1093/nar/gkz114 | RRID:SCR_016945 |  |
| software, algorithm | VSN in DESeq2 | Bioconductor  doi:10.1093/bioinformatics/18.suppl_1.S96, doi:10.1186/s13059-014-0550-8 | RRID:SCR_015687 |  |
